# Providing Support Differentially Affects Asian American and Latinx Psychosocial and Physiological Well-Being: A Pilot Study

**DOI:** 10.3389/fpsyg.2022.869715

**Published:** 2022-05-26

**Authors:** Shu-Sha Angie Guan, Gabriela Jimenez, Jennifer Cabrera, Anna Cho, Omar Ullah, Ruben Den Broeder

**Affiliations:** ^1^Department of Child and Adolescent Development, California State University, Northridge, CA, United States; ^2^Department of Environmental and Occupational Health, California State University, Northridge, CA, United States; ^3^Department of Health Sciences, California State University, Northridge, CA, United States

**Keywords:** providing support, expressive helping, self-esteem, stress, TSST

## Abstract

Although substantial evidence suggests receiving social support has positive implications for well-being, less is known about how providing support can confer benefits, particularly for Asian American and Latinx individuals who are more likely to come from interdependent cultures that emphasize family obligation. Asian American and Latinx college students (*N* = 48; *M*_age_ = 21.44, *SD* = 2.61; 68.75% female) reported on anxiety before taking part in a modified laboratory task that elicited a physiological stress response as measured by total cortisol output. They were randomly assigned to write (a) a supportive note to a family member, (b) a supportive note to a close friend, or (c) about their day in a control condition after the mild lab stressor and reported on psychosocial well-being (i.e., post-task anxiety and self-esteem). Those who provided support to a family member experienced higher self-esteem compared to those in the control condition. However, there was variation in Asian American and Latinx participants’ physiological stress response (i.e., total cortisol output). The findings suggest that providing support to close others, particularly family members, can be differentially meaningful for individuals from diverse backgrounds.

## Introduction

**Figure 1:**
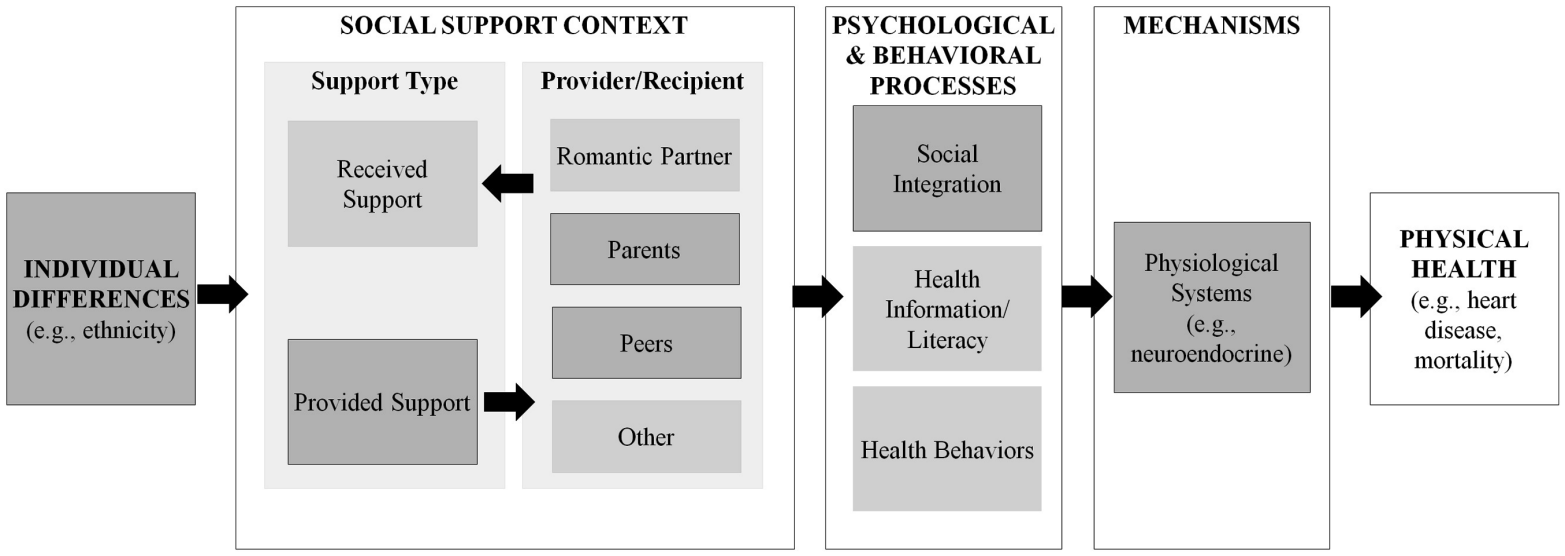
Conceptual model of potential pathways to physical health outcomes. Darkened boxes are constructs examined in the current study.

A growing literature suggests a brain–body connection such that psychological and behavioral experiences can “get under the skin” to affect biological functioning and physical health. As shown in Figure 1, positive social experiences, such as receiving social support from family and friends when in need, can have a powerful effect on psychological (e.g., feeling socially integrated, high self-esteem, and low depressive symptoms) and physiological functioning (e.g., neuroendocrine system response to stressors) in ways that ultimately shape the development of disease (e.g., McEwen and Seeman, 1999; Seeman et al., 2001; Thoits, 2011; Uchino et al., 2019). For example, a meta-analysis of 148 studies suggests that feeling socially connected can reduce the risk of pre-mature mortality at rates comparable to quitting smoking or drinking independent of other risk factors, such as high body mass index (BMI) and a sedentary lifestyle (Holt-Lunstad et al., 2010).

Receiving support can modify the effect of negative experiences on physiological stress response systems like the hypothalamic pituitary adrenal (HPA) axis. As part of the neuroendocrine system, the HPA axis can be assessed through the measurement of the hormone end-product cortisol and is highly sensitive to social experiences. It is believed to be activated by acute and chronic stressors, such as social-evaluative threats (McEwen and Seeman, 1999; Dickerson and Kemeny, 2004; Dickerson et al., 2008). Although HPA response to an immediate stressor can be adaptive short-term in helping individuals deal with the stressor, repeated exposure can dysregulate the neuroendocrine system resulting in dampened ability to respond to future stressors or hyperactivity and heightened cortisol output (e.g., McEwen and Seeman, 1999; Seeman et al., 2001). Positive social experiences like social support can disrupt this process by reducing activation or aiding in quicker recovery of the HPA axis in ways that are protective and, ultimately, decrease disease vulnerability long-term (Uchino et al., 2019).

Structural and functional aspects of social experiences can have direct and stress-buffering effects on health (for reviews, see: Cohen and Wills, 1985; Thorsteinsson and James, 1999; Thoits, 2011). For example, providing “emotional sustenance” or support (e.g., empathic understanding) or active coping assistance (e.g., informational support like advice, instrumental support like financial aid) can reduce arousal in psychological and physiological stress response systems like the HPA axis (Thorsteinsson and James, 1999; Thoits, 2011; Uchino et al., 2019). However, there are many contexts in which receiving support may not be helpful. For example, advice that is unsolicited or unsupportive can be detrimental or threatening to one’s sense of self-efficacy (Beehr et al., 2010; Uchino et al., 2011), particularly within cultures concerned about the loss of “face” or disruption of social harmony (Kim et al., 2008; Wang and Campos, 2017).

Although much of the health literature has focused on received support, providing support can also be uniquely rewarding (e.g., Inagaki and Eisenberger, 2016; Inagaki and Orehek, 2017; Inagaki, 2018). For example, providing help through charitable donations elicits activation in reward regions of the brain in similar ways to receiving monetary rewards for oneself (Moll et al., 2006). Additionally, older adults who participate in volunteer programs often report that volunteering contributed to their communities, improved their lives, and helped them feel better about themselves (e.g., Morrow-Howell et al., 2009). The benefits may be particularly robust for seniors from low educational and socioeconomic status backgrounds, suggesting that engaging in service can be an empowering social experience. Although attenuated, there are benefits to life satisfaction and perceived health for young adults who volunteer relative to their non-volunteer counterparts as well (e.g., Van Willigen, 2000).

Providing social support can also have implications for physiological health (Inagaki, 2018). For example, writing a supportive note to a close friend can reduce stressor-induced sympathetic nervous system response relative to a control group among a sample of young adults from diverse backgrounds (Inagaki and Eisenberger, 2016). Although the aforementioned study found no differences in psychological stress or cortisol reactivity across conditions, research using this expressive helping paradigm has focused on providing support to a friend or imaginary stranger rather than family member (e.g., other patients who will be preparing for surgery; see also Rini et al., 2014; Williamson et al., 2017). Additionally, expressive helping work has focused on non-Hispanic White populations when the effects of social relationships on well-being can vary across context and culture (e.g., Taylor et al., 2007; Kim et al., 2008; Uchino et al., 2011; Guan and Fuligni, 2016; Guan et al., 2017).

Providing assistance may be particularly important in creating a sense of community or fulfilling a sense of family obligation for youth from interdependent Asian and Latin American cultural backgrounds (e.g., Fuligni et al., 1999; Telzer and Fuligni, 2009). Relative to individuals from independent cultural backgrounds, individuals from interdependent cultural backgrounds place greater value on group goals and family obligation, a sense of duty to assist and to take into account the needs and wishes of the family when making decisions (Fuligni et al., 1999). These values may manifest in various ways. On one hand, Asian American and Latinx young adults may be less likely to seek help or benefit affectively and physiologically from received, explicit support compared to their European American counterparts given the emphasis on social harmony (e.g., Kim et al., 2008; Guan and Fuligni, 2016; Wang and Lau, 2018). On the other hand, they find great value in family relationships and providing assistance (e.g., Taylor et al., 2007; Telzer and Fuligni, 2009). Youth from Asian American and Latinx backgrounds often report higher levels of doing chores, taking care of siblings, translating and interpreting, and spending time with their families than their European American counterparts (Fuligni et al., 1999). Therefore, while family assistance and relationships can be a source of stress (e.g., Guan et al., 2020), it can also be protective (Taylor et al., 2007; Wang and Campos, 2017; Campos et al., 2018). Indeed, on days in which youth provide assistance to their families, they reported more feelings of happiness (psychological well-being) and this was explained in large part by a higher sense of role fulfillment (e.g., feeling like a good son/daughter; Telzer and Fuligni, 2009). Altogether, this research suggests that providing social support rather than receiving support will be more beneficial in stress reduction for young adults from Asian American and Latinx backgrounds.

In the current study, we hypothesize that (a) providing social support to a close family member or friend will confer greater psychological (i.e., self-esteem) and physiological (i.e., reduced cortisol output) benefits for Asian American and Latinx young adults experiencing stress relative to a control group where no support is given; additionally, (b) providing support to a family member will be more beneficial in terms of reducing stress and feeling good about oneself relative to providing support to a friend. In testing these hypotheses, this novel, pilot study seeks to further our understanding of how culture can shape socioemotional experiences in ways that affect psychological and physiological health.

## Materials and Methods

### Participants and Procedure

College students (*N* = 48; *M_age_* = 21.44, *SD* = 2.61; 68.75% female) from Asian American (*n* = 18; majority East Asian, followed by Southeast Asian, Pacific Islander, and South Asian) and Latinx backgrounds (*n* = 30; majority Mexican American, followed by Central American and South American) from a large, public university in Southern California were recruited *via* campus flyers. Healthy students (e.g., who did not have a viral infection in the last 24 h or known inflammatory, endocrine, or cardiovascular condition) aged 18 years or older who were not pregnant were scheduled in groups (1–3 participants) for lab sessions between the hours of 12 and 6 pm to account for the diurnal rhythm of cortisol. Asian American participants reported higher average father’s education (between technical or trade school to community college) than Latinx participants (between secondary or high school education and technical or trade school), *t*(45) = 2.14, *p* = 0.038.

**Figure 2:**
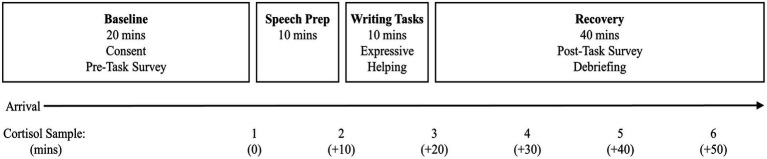
Experimental and cortisol collection timeline.

As shown in Figure 2, after a brief survey and resting period, participants completed a mock Trier Social Stress Test (TSST; Kirschbaum et al., 1993) in which participants were guided to an observation room with a one-way mirror and video camera. They were instructed by an experimenter to prepare for a mild stressor (i.e., public speech in which they describe why they are the ideal candidate for their dream job) for 10 min. Participants were scheduled in groups to elicit the presence of an evaluative audience and potential for social comparison (Dickerson and Kemeny, 2004; Von Dawans et al., 2011). Rather than completing the speech and original math task, participants were told that, due to equipment failure, they would complete a 10-min writing task instead. Participants wrote about a stressful experience and were randomly assigned to provide (a) a supportive letter to a close family member who might be experiencing a similarly stressful experience (*n* = 16), (b) a supportive letter to a close friend who might be experiencing a similarly stressful experience (*n* = 19), or (c) a list of the activities of their day (control group; *n* = 13) based on an expressive helping paradigm (Rini et al., 2014). Specifically, instructions for the experimental conditions were as follows:

**Family Support Condition**. *Name a non-romantic family member you are close to. What is your relationship? Write about a personally upsetting experience and describe the feelings you had about the experience (5 min). Your family might benefit from learning about your experiences and strategies in coping with the emotions from this challenging or stressful situation. On the next page, write as if you are speaking to the close family member you named experiencing the same upsetting emotions (5 min).*

**Friend Support Condition**. *Name a non-related, non-romantic friend you are close to. How long have you known them? Write about a personally upsetting experience and describe the feelings you had about the experience (5 min). Your friends might benefit from learning about your experiences and strategies in coping with the emotions from this challenging or stressful situation. On the next page, write as if you are speaking to the close friend you named experiencing the same upsetting emotions (5 min).*

**Control (No Support) Condition**. *Enter today’s date and time. Write about a personally upsetting experience and describe the feelings you had about the experience (5 min). On the next page, please write about what you did today (5 min).*

This expressive helping writing task also serves as an emotion induction procedure designed to continue eliciting a negative affective state and stress response as an alternative to cognitive and verbal interaction tasks, such as the mental math task in the original TSST (Dickerson and Kemeny, 2004; Dickerson et al., 2008). In a meta-analysis of acute stress tasks that elicit cortisol increases, Dickerson and Kemeny (2004) found that anticipation of a psychological challenge is sufficient to elicit a physiological stress response and tasks that induce social evaluative threat, including potential recordings for subsequent evaluation, presence of an evaluative audience other than the experimenter, and presence of potential out-performance by another participant, were characteristics in laboratory tasks that significantly increased cortisol levels. We expect cortisol levels to decrease 20–30 min after the expressive writing task.

After the writing task, participants completed a post-task survey and rested quietly until the end of the session. Throughout the session, participants provided six saliva samples with cotton Salivettes (Sarstedt, Rommelsdorf, Germany) every 10 min. Samples were stored at −20 °C until overnight delivery over ice to Biochemisches Laboratory, Universitat Trier, Germany to be assayed in duplicate for cortisol. After thawing, cortisol levels were determined using a solid phase time-resolved fluorescence immunoassay with flouromeric end-point detection (DELFIA). The average inter-assay coefficient of variation was 7.0%.

### Measures

#### Psychological Stress

The short-form State-Trait Anxiety Inventory (STAI; Marteau and Bekker, 1992) assessed participants’ immediate feelings of stress (e.g., “I feel upset,” “I feel worried”) before the stress and writing tasks (pre-task survey) and immediately after the tasks (post-task survey) on a 4-point scale (*αs* = 0.77, 79).

#### Physiological Stress

Cortisol levels were log-transformed because they demonstrated positive skew (1.211–2.21, *SE* = 0.34) and kurtosis (1.15–7.62, *SE* = 0.67). Total area under the curve (AUC) with respect to ground was calculated based on equations outlined in Pruessner et al. (2003). As discussed in Pruessner et al. (2003), given multiple cortisol measurements but limited sample size, AUC measures maximize power without sacrificing information and has been used to assess the effect of an intervention over an entire session in clinical trials.

#### Self-Esteem

The Rosenberg self-esteem scale (Rosenberg, 1965) assessed post-task self-esteem with items such as “I feel I have a number of good qualities” and “I feel I do not have much to be proud of (reverse-coded)” on a 5-point scale (*α* = 0.92).

#### Body Mass Index

Body mass index (BMI) was calculated using participant-reported height and weight, and based on the Centers for Disease Control and Prevention formula where weight in pounds (lbs) was divided by squared height in inches and multiplied by 703 to capture adiposity and index obesity risk which has been shown to affect cortisol secretion. In the current study, there were no differences in BMI between Asian American (*M* = 22.99, *SD* = 4.21) and Latinx participants (*M* = 24.32, *SD* = 4.77), *t*(45) = −1.00, *p* = 0.343. In addition to demographic differences, BMI is a covariate indicator of general health used in previous studies examining HPA function (Adam and Kumari, 2009).

## Results

### Psychological Stress

A 2 (timing: pre vs. post) × 3 (condition) mixed ANCOVA controlling for ethnicity showed significant change in self-reported psychological stress from pre- (*M* = 1.68, *SD* = 0.08) to post-task (*M* = 1.98, *SD* = 0.09) which suggests the manipulation was effective, *F*(1,42) = 18.18, *p* < 0.001, *η*^2^ = 0.302. However, there was no timing by condition interaction, *F*(2, 42) = 1.41, *p* = 0.256, *η*^2^ = 0.063, which suggests no change in psychological stress across the three conditions. Ethnicity did not moderate these results (*ps* > 0.05).

### Physiological Stress

**Figure 3:**
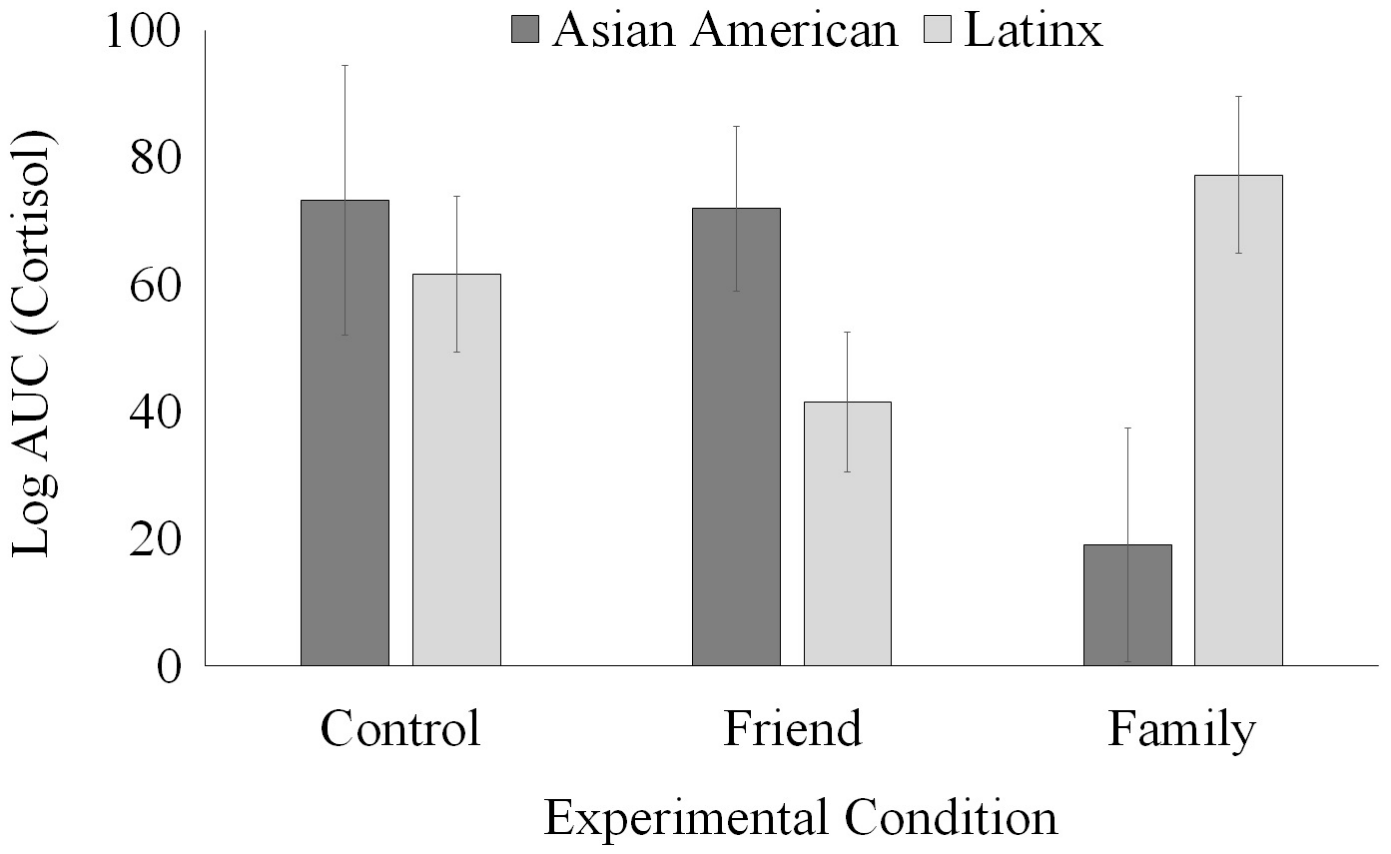
Total salivary cortisol response by condition and ethnicity.

A 2 (ethnicity: Asian American vs. Latinx) × 3 (condition) ANCOVA controlling for BMI was conducted to examine differences in AUC. As shown in Figure 3, there was a significant condition by ethnicity interaction (*F*(2, 38) = 5.43, *p* = 0.008, *η*^2^ = 0.222). *Post hoc* Bonferroni comparisons revealed that Asian American participants who provided support to a close family member (*M* = 19.03, *SE* = 18.33) showed significantly lower AUC cortisol output compared to their Latinx counterparts (*M* = 77.21, *SE* = 12.32), *p* = 0.01. On the other hand, Latinx participants who provided support to a friend (*M* = 41.44, *SE* = 10.97) showed marginally lower AUC cortisol output compared to their Asian Americans counterparts (*M* = 71.93, *SE* = 21.95), *p* = 0.079. Asian American (*M* = 73.25, *SE* = 21.25) and Latinx (*M* = 61.58, *SE* = 12.27) participants in the control group did not differ in AUC (*p* = 0.632).

### Self-Esteem

A 2 (ethnicity: Asian American vs. Latinx) × 3 (condition) ANCOVA controlling for BMI revealed a significant main effect of condition on self-esteem, *F*(2, 40) = 3.26, *p* = 0.049, *η*^2^ = 0.140. *Post hoc* Bonferroni comparisons showed that young adults who provided support to a family member reported significantly higher self-esteem (*M* = 3.17, *SE* = 0.18) relative to those in the control condition (*M* = 2.48, *SE* = 0.19; *p* = 0.046). There were no significant differences in self-esteem between those who provided support to a family member relative to a friend (*M* = 2.89, *SE* = 0.15; *p* = 0.74) or provided support to a friend relative to those in the control group (*p* = 0.24). There was also no significant main effect or interaction by ethnicity (*ps* > 0.05).

## Discussion

Given research that suggests social relationships can affect psychosocial well-being and “get under the skin” to affect physical health (e.g., Cohen and Wills, 1985; Uchino et al., 2019) and that the effect may be particularly important for individuals from cultural backgrounds that place greater value on family obligation (e.g., Fuligni et al., 1999), we hypothesized that providing support to family members and, to a lesser extent, friends would reduce psychological and physiological stress among young adults from Asian American and Latinx backgrounds. Overall, participants who provided support to a family member reported significantly higher self-esteem relative to the control group, supporting the idea that individuals from Asian American and Latinx backgrounds find meaning and feel good about themselves through helping family members (Fuligni et al., 1999; Telzer and Fuligni, 2009).

Our results also partially supported the differential physiological implications of providing support to a family member relative to a friend, even a close one. Asian American young adults who provided support to a close family member showed significantly lower AUC relative to their Latinx counterparts; however, Latinx young adults who provided support to a close friend showed marginally lower physiological stress relative to their Asian American counterparts. These findings complement research that suggests various types of relationships (e.g., “intimate and enduring” strong ties; Thoits, 2011) may be differentially important to shaping health outcomes for individuals from diverse cultures. Although there is evidence that the experimental manipulation used in the current study (i.e., mock, group TSST) was effective in increasing self-reported psychological stress, there were no differences across support conditions in psychological stress reactivity (i.e., change in state anxiety) and this is line with previous research (e.g., Telzer and Fuligni, 2009; Inagaki and Eisenberger, 2016).

Although all participants experienced greater self-esteem in providing help to their families, differences in socioeconomic status and group identity may contribute to ethnic differences in how various social group members (i.e., family relative to friends) affect physiological stress response. For example, Latinx participants, who reported lower parental education levels (one indicator of socioeconomic status), may have been primed to think about various other challenges faced by their families. Additionally, although Asian American and Latinx youth both report greater family obligation relative to their European American counterparts (e.g., Fuligni, 1998; Fuligni et al., 1999), Latinx youth report spending more time assisting the family and being less likely to disagree with parents (Fuligni, 1998; Telzer and Fuligni, 2009). This family deference and caregiving burden may violate the ecological validity of the expressive helping paradigm as a family support provision task and attenuate the stress-buffering effects of the family support condition (Inagaki and Orehek, 2017; Inagaki, 2018). Ethnic differences in physiological stress response may also reflect greater identification with peers compared to family members by Latinx participants (Thoits, 2011). Future research should examine how alternative operationalizations of support provision can affect various physiological systems (e.g., monetary contributions on neural reward activity; Telzer et al., 2010).

The effectiveness of the support provision paradigm on mechanisms linked to physiological well-being varied by ethnicity and this provides a possible explanation for why previous studies on expressive helping to a friend or stranger did not produce significant differences in neuroendocrine response (e.g., Inagaki and Eisenberger, 2016). Additionally, the disconnect between survey or behavioral measures compared to objective physiological measures is consistent with prior research (Taylor et al., 2007; Telzer et al., 2010; Guan et al., 2017) and suggests that physiological processes may capture what is not self-reportable. These results highlight the need for future research to incorporate and assess multiple biomarkers in psychosocial research, particularly with subjective experiences such as stress and among populations that may have alternative reporting norms surrounding mental and physical health.

Although these pilot results are promising, a few notable limitations must be addressed. This was a small sample of majority female college students and the characteristics of the sample likely limited our analytic power and ability to conduct moderation analyses. As a result, these pilot results should be interpreted with caution. Additionally, instead of using ethnicity as a proxy for cultural differences, future research should explore how specific cultural values (e.g., *familism,* family role fulfilment) affect stress and cortisol reactivity (e.g., Telzer et al., 2010; Wang and Campos, 2017).

Despite limitations, the changes in cortisol observed in the current study are comparable to prior studies assessing cultural differences in stress reactivity using the TSST (e.g., Taylor et al., 2007; Guan et al., 2017) and the findings have important implications. They indicate potentially positive benefits and pride in being able to assist close others for young adults from cultural backgrounds that emphasize strong interdependent social relationships. The results also emphasize the need for future research to include minority populations and incorporate multiple measures of subjective, culture-variant constructs such as stress and health (e.g., self-report and biological markers). Giving behaviors can be actor-initiated and controllable (Inagaki, 2018), and this study further highlights the promise of focusing on support provision in the development of culturally-responsive health interventions.

## Data Availability Statement

The data used/or analyzed in the current study are available from the corresponding author upon reasonable request.

## Ethics Statement

This study was reviewed and approved by the Committee for Protection of Human Subjects at California State University, Northridge. The participants provided their written informed consent to participate in this study.

## Author Contributions

SG contributed to developing the study, data analysis, writing, and managing the manuscript submission process. GJ contributed to data collection, managing the measures, writing, and editing the manuscript. JC, AC, and OU contributed to data collecting, writing, and editing the manuscript. RB contributed to writing and editing the manuscript. All authors contributed to the article and approved the submitted version.

## Funding

This study was supported by the American Psychological Association (APA) Promoting Psychological Research and Training on Health Disparities Issues at Ethnic Minority Serving Institutions (ProDIGs) Grant. This work was also supported by NIH NIGMS RL5GM118975 and TL4GM118977.

## Conflict of Interest

The authors declare that the research was conducted in the absence of any commercial or financial relationships that could be construed as a potential conflict of interest.

## Publisher’s Note

All claims expressed in this article are solely those of the authors and do not necessarily represent those of their affiliated organizations, or those of the publisher, the editors and the reviewers. Any product that may be evaluated in this article, or claim that may be made by its manufacturer, is not guaranteed or endorsed by the publisher.
